# An educational program, «Women victims of domestic violence: Detection, clinic, help»: Working with the complexity of teaching and Interpreting practice through research

**DOI:** 10.1192/j.eurpsy.2024.1678

**Published:** 2024-08-27

**Authors:** N. D. Semenova, M. A. Kachaeva, S. V. Shport

**Affiliations:** ^1^Laboratory of Psychological Counseling, Moscow Research Institute of Psychiatry – a branch of V. Serbsky National Medical Research Centre for Psychiatry and Narcology; ^2^Department of forensic psychiatric examination; ^3^Administration, V. Serbsky National Medical Research Centre for Psychiatry and Narcology, Moscow, Russian Federation

## Abstract

**Introduction:**

Firstly, we will speak on the violence against women from a Russian perspective. The selected reports from regional psychiatric services and police department reports of domestic violence cases will be presented.

**Objectives:**

Secondly, we will draw upon our work developing and providing a new educational program, «Women victims of domestic violence: Detection, clinic, help,» mainly based on teaching several modules, WPA International Curriculum for Mental Healthcare Providers on Violence Against Women.

**Methods:**

In this present paper, we examine evidence-based practice from the starting points of research as illumination and psychiatry as a discipline with hermeneutic potential, to consider relationships between research and practice and the opportunities available within the current research agenda for psychiatrists and clinical psychologists working in clinical settings.

**Results:**

We contend that the quality of women’s mental health services will only improve when they can acknowledge the considerable impact that intimate partner violence and sexual violence, as well as social inequalities, especially those based on gender, have on women’s mental health. We do not underestimate the difficulty of providing practical help to women whose mental health has been profoundly affected by the violence, damage that is often further compounded by years of mistreatment and revictimization in services.

**Conclusions:**

The paper provides commentaries and reflections on the steps that must be taken to create opportunities to foster dialogue, discussing and exchanging ideas on a diverse range of topics relevant to the advancement of the program in the broader context.

**Disclosure of Interest:**

None Declared

